# Peripheral Arterial and Venous Response to Tilt Test after a 60-Day Bedrest with and without Countermeasures (ES-IBREP)

**DOI:** 10.1371/journal.pone.0032854

**Published:** 2012-03-07

**Authors:** Ming Yuan, Mickael Coupé, Yanqiang Bai, Guillemette Gauquelin-Koch, Shizhong Jiang, Patrick Aubry, Yumin Wan, Marc-Antoine Custaud, Yinghui Li, Philippe Arbeille

**Affiliations:** 1 State Key Laboratory of Space Medicine Fundamentals and Application, China Astronaut Research and Training Center, Beijing, China; 2 Unité Mixte de Recherche, Centre National de la Recherche Scientifique, Institut National de la Santé et de la Recherche Médicale, Faculté de Médecine d'Angers, Angers, France; 3 Centre National d'Etudes Spatiales, Paris, France; 4 UMPS-CERCOM Médecine Physiologie spatiale, Universite-Hôpital Trousseau, Tours, France; Institut Pluridisciplinaire Hubert Curien, France

## Abstract

We quantified the impact of 60-day head-down bed rest (HDBR) with countermeasures on arterial and venous response to tilt. Methods: Twenty-one males: 7 control (Con), 7 resistive vibration exercise (RVE) and 7 Chinese herb (Herb) were assessed. Subjects were identified as finisher (F) or non-finishers (NF) at the post-HDBR 20-min tilt test. The cerebral (MCA), femoral (FEM) arterial flow velocity and leg vascular resistance (FRI), the portal vein section (PV), the flow redistribution ratios (MCA/FEM; MCA/PV), the tibial (Tib), gastrocnemius (Gast), and saphenous (Saph) vein sections were measured by echography and Doppler ultrasonography. Arterial and venous parameters were measured at 3-min pre-tilt in the supine position, and at 1 min before the end of the tilt. Results: At post-HDBR tilt, MCA decreased more compared with pre-HDBR tilt in the Con, RVE, and Herb groups, the MCA/FEM tended to decrease in the Con and Herb groups (not significant) but remained stable in the RVE gr. FRI dropped in the Con gr, but remained stable in the Herb gr and increased in the RVE gr. PV decreased less in the Con and Herb groups but remained unchanged in the RVE gr. MCA/PV decreased in the Con and Herb groups, but increased to a similar extent in the RVE gr. Gast section significantly increased more in the Con gr only, whereas Tib section increased more in the Con and Herb groups but not in the RVE gr. The percent change in Saph section was similar at pre- and post-HDBR tilt. Conclusion: In the Con gr, vasoconstriction was reduced in leg and splanchnic areas. RVE and Herb contributed to prevent the loss of vasoconstriction in both areas, but the effect of RVE was higher. RVE and Herb contributed to limit Gast distension whereas only RVE had a protective effect on the Tib.

## Introduction

Several spaceflight and bedrest studies have reported a significant lack of increase in vascular resistance at the leg level and or a lack of reduction in portal flow in response to fluid shift downwards towards the feet as provoked by stand, lower-body negative pressure (LBNP) or tilt tests [1], [2]. Such observations have been correlated with orthostatic intolerance and interpreted as a deficit in vasoconstriction in these territories (lower limb and splanchnic areas). Moreover, such deficits in leg arterial vasoconstriction were not related to a reduction in sympathetic activity as measured by micro-neurography in the peroneal nerve but to regional vascular responsiveness [3]. Conversely, an exaggerated increase in tibial and gastrocnemius vein sections during tilt, LBNP or stand tests was found in non-finisher (NF) subjects after head-down bedrest (HDBR) [4]. Only aerobic exercise coupled to LBNP (countermeasure (CM)) was found to efficiently prevent the lack of vasoconstrictive responses at the leg and splanchnic levels and also the increase in leg vein distensibility after HDBR [2], [4]. Despite their efficiency, such CMs are time-consuming and are not necessarily adapted for preventing the degradation of other systems such as the neurosensorial system (otolith, muscle proprioceptors, three-dimensional visual reference) which act on the distal vasomotricity and may also limit the capacity of the subject to stand, walk, exercise, and control his/her posture [5]. The CM that looks the most adapted is artificial gravity as obtained with a short-arm centrifuge, the head being on the rotating axis and the feet at the extremity of the rotating arm [6]. However, such CMs require the development of complex and heavy devices to enable use in a space habitat.

Other mechanical CMs such as resistive vibration exercise (RVE) are very simple to use and not time-consuming. They have been found to be efficient against bone loss and muscle atrophy [7], [8], but their effect on cardiac and peripheral vascular targets is not very clear. Some bedrest studies reported that foot vibration induces increases in sympathetic activity and repeated vasoconstriction in the lower limbs and partially prevents the drop in blood pressure during the stand test [9], [10]. Moreover, RVE has been found to stimulate the neuroendocrine and neuromuscular systems [11]. Recently, some studies showed that RVE has direct effects on the remodeling of the structure and function of vascular tissue. van Duijnhoven et al. [12] reported that 60 days of 6° head-down tilt bedrest deconditioning resulted in large arterial adaptations in a group of young males, and that RVE effectively attenuated the reduction in the diameter of the conduit artery and preserved endothelial function to bed rest.

In China, traditional Chinese medicine (TCM) has been regarded as a potential CM for adverse physiological effects induced by spaceflight or bedrest. Chinese herb CMs that affect several metabolic functions have been found to be appropriate for partially preventing the effect of aging and immobility [13]. Herb pills made of ginsenosides and ligustrazine have been reported to have beneficial effects on the endothelium by promoting the synthesis of nitric oxide (NO) [14] and relaxation of resistance vessels [15]. *Astragalus mongholicus* has also been reported to enhance NO production via endothelial nitric oxide synthase (eNOS) activation and scavenging of reactive oxygen species (ROS) [16]. One report showed that herbs may have direct effects on vascular function. For example, *Schisandra chinensis* extract has relaxant effects upon endothelium-intact and denuded aortas which are mediated by the NO pathway on the endothelium and by dephosphorylation of myosin light chain (MLC) on vascular smooth muscle [17].

Our hypothesis was that (a) daily RVE may reduce the deleterious effect of HDBR on the peripheral arterial and venous response to the tilt-induced fluid shift; (b) daily ingestion of Chinese herbs may improve or maintain the response of various arterial and venous targets to tilt by reducing the HDBR effect. The objective of the present study was to quantify the impact of a 60-day HDBR with the CMs cited above on the arterial and venous response to tilt test.

## Materials and Methods

All subjects received a complete description of the experimental procedure before giving their written informed consent to be included in the study. The protocols conformed to Helsinki declaration and were approved by the ethical committee of China Astronaut Research and Training Center (ACC, Beijing, China).

Twenty-one healthy male subjects participated in a 60-day −6° HDBR at the China ACC. Before HDBR, the subjects were aged 30.4±0.9 years, with average weights and heights of 59.9±0.9 kg and 168.5±0.7 cm, respectively. None had a history of cardiovascular or other major diseases. All subjects underwent an extensive medical examination before being included in this study. The subjects were randomly assigned into control group “Con gr” (HDBR without CM, n = 7), HDBR with daily consumption of Chinese herbs (Herb gr, n = 7) and HDBR with daily resistive vibration exercise (RVE gr, n = 7).

### HDBR program

The study consisted of a 15-day ambulatory control period, 60-day HDBR and 24-day recovery period. During bedrest, the subjects remained in a −6° head-down tilt position continuously except a daily 10-min stand for hygienic procedures and weighting. Coffee, tea and smoking were prohibited throughout the experiment. The subjects were supervised and monitored 24 h per day. Room lighting was on between 6:30 am and 10:30 pm. Subjects in the Herb gr were given orally 6-g pills, 3 times daily. The herb pills were made according to the Tai Kong Yang Xin Chinese herb prescription (TKYXCHP), which include ≈10 Chinese herbs. The main parts of TKYXCHP are Panax ginseng, *Astragalus membranaceus*, *Ligusticum wallichii*, *Schisandra chinensis*, *Radix ophiopogonis*, prepared Rehmania root, *Rhizoma drynariae*, and *Poria cocos*. The placebo was given orally to control subjects based on the double-blind principle. The subjects in the RVE gr were exposed to a once-daily session of RVE for 24 min according to the training protocol (i.e., each session consisted of five stretches, with 4-min RVE each and with 1-min rest). The characteristics of the RVE platform scenario were: 30-Hz vibration, resistive loads of 1.5-times the body weight, magnitude of 0.3 g peak-to-peak, and amplitude <0.1 mm.

### Blood sample

Fasting blood samples were taken by the ACC staff between 6:30 and 7:00 AM from the antecubital vein of the left arms in the ambulatory control phase(day 11 before HDBR) and during the last day of HDBR(day 60). Haemoglobin concentration and the hematocrit values were measured at once by blood cell counter and capillary tubes microhematocrit centrifuger according to the recommendations of international council for standardization in haematology(ICSH) [18], respectively. The samples added with EDTA-Na2 and aprotinin were centrifuged at 3000 RPM at 4°C for 10 min, the plasma was isolated and stored at minus 80°C until analysis.

Atrial Natriuretic Peptide(ANP) and AngiotensinII(AngII) were quantified using Radioimmunoassay(RIA) from Atom High Tech Corporation(Beijing, China, Cat No. IMK- 0459) and FuRui Bio-engineering Corporation(Beijing, China, Cat No. FR-FJ-026), respectively. Norepinephrine(NE) and Epinephrine(E) were determined using ELISA kits purchased from SunBio(Beijing, China, Cat No J-0004 and J-0010). All of the instructions from the purchased assay kits were followed without deviation.

The changes in plasma volume(ΔPV) were determined using the Dill & costill equation, validated for bed-rest by Johansen et al [19]:
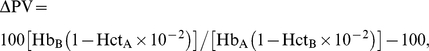
where Hb are haemoglobin values and Hct are hematocrit values, measured before(suffix “B”) and after(suffix “A”) HDBR. Hb and Hct were measured on venous blood samples taken from the antecubital vein of the left arm [20].

### Tilt test scenario

Each subject participated in a tilt test at the pre- (4 days before HDBR) and post-HDBR (first stand up at the end of HDBR). The tilt test consisted of 10 min of rest in the supine position for instrumentation followed by 10 min of resting measurements, then a 75° head-up tilt for ≤20 min. During these tests, HR was obtained by three-lead electrocardiography (GE Healthcare, Milwaukee, WI, USA). Systolic blood pressure (SBP) and diastolic blood pressure (DBP) were measured continuously with Cardiopres, a non-invasive finger cuff method (Biomedical Instrumentation TPD-TNO, Amsterdam, the Netherlands). A height corrector placed at the level of the heart provided stable measurements independent of hand movement, position or tilting. The test was ended if one of the following signs occurred: pre-syncope symptoms (nausea, clammy skin, excessive sweating, pallor, vertigo), SBP fall >25 mmHg/min or DBP fall >15 mmHg/min, a SBP<70 mmHg, heart rate (HR) fall >15 beats per min, or cardiac dysrhythmias. On the basis of these criteria, subjects were identified as “finishers” (F) and “non-finishers” (NF) at post-HDBR 20-min tilt test.

### Echographic and Doppler measurements

During the tilt test, the cerebral flow velocity (middle cerebral artery (MCA) flow) was recorded using a 2-MHz transcranial Doppler probe fixed over the temporal window to insonate the right MCA. The angle of insonation of the MCA was considered to be 0°. The velocity of flow of the superficial femoral artery (FEM) was investigated using a flat Doppler probe of 4 MHz fixed by two straps passing around the upper part of the thigh and around the abdomen. The Doppler beam was steered at 45° from the front face of the probe, and the angle between the Doppler beam and the vessel axis remained unchanged during the session. The Doppler spectrum was recorded and analyzed by the Cardiolab Ground Module (CNES, Paris, France). Based on an earlier report [21], it was assumed that the diameter of these vessels remained constant during the tilt test, and that the mean velocity changed in proportion with flow volume (mL/min) as calculated from the velocity and cross-section of the vessel. The portal vein cross-section area (PV) was measured on a transverse view of the portal vein trunk by a sonographer handling the probe and collecting data during the tilt test approximately at the intersection of the mammary and xiphoid lines (lower external quarter). It was considered that changes in flow in the portal vein reflected changes in the cross-sectional area (CSA) [2].The tibial vein(Tib), gastrocnemius vein(Gast) and saphenous vein(Saph) were investigated according to a transverse view thanks to a T-shaped flat echographic probe fixed on the skin at the upper internal part of the calf. Blood pressure (BP) was measured by arm cuff and Cardiopres.

### Parameter display

Cerebral (MCA) and femoral (FEM) flow velocity were measured continuously by Doppler during the tilt test. BP was also measured by arm cuff and Cardiopres. Portal-vein flow (PV) was measured every 3 min by the sonographer during the tilt. All these parameters were measured at 3-min pre-tilt in the supine position at (−1 min), before the end of the test. The percent changes were displayed as mean±SD.

### Statistical analyses

The percent change from supine to 1 min before the end of the tilt test were analyzed with data grouped according to the CMs used (RVE, Herb). Data are mean±SD. Statistical comparisons were made using two-way repeated measures analyses of variance (SAS 9.1.3 analysis software; Cary, NC, USA) with the main effect of the CMs at post-HDBR 20-min tilt. P<0.05 was considered significant.

## Results

There was increasing tendency of body weight in Con gr and Herb gr, but not in RVE gr after 60 d HDBR, HR increased significantly in Con gr(P<0.001, Table 1), but decreased or maintained stable in RVE gr and Herb gr. There was no significant difference of DBP after 60 d HDBR compared with before values in all groups. There was increasing tendency of plasma NE in all groups, but the difference didn't reach the statistical significance. After 60 d HDBR, plasma E and AngIIincreased significantly in Con gr and Herb gr, but not in RVE gr. Compared with before HDBR, ANP increased significantly in all groups. There was not statistical significance of the difference in the changes of plasma volume after HDBR among three groups.

**Table 1:** Subject characteristics, blood biochemical parameters and changes in plasma volume before and after 60 days of HDBR without(Con) or with countermeasures (RVE and Herb).

Parameters	Con		RVE		Herb	
	Before	After	Before	After	Before	After
Age(years)	30.3±3.2	/	30.7±5	/	30.3±5.5	/
Height(cm)	169±4.4	171±4.7***	168±3.0	168±3.2	168±4.1	169±5.1
Weight(kg)	63.1±2.7	64.5±1.97	62.7±5.9	60.7±5.1	60.7±5.4	63.1±6.7
HR(bpm)	69.1±6	76.9±6.3***	72±5.8	68.6±7.7	73.9±8.5	73.1±8.8
SBP(mmHg)	114±6.9	109±8.5	118±6.6	110±5.1*	117±3.8	115±4.6
DBP(mmHg)	68±6.2	69±9.7	72±4.2	69±3.8	70±5.4	75±4.7
NE(pmol/L)	303.3±40	315.9±65	291.8±38	333.5±60	284.4±84	333.1±66
E(pg/ml)	39.8±22	76.8±35.7*	69.7±33	71.1±26	48±16.3	87.6±32.2*
ANP(ng/ml)	0.4±0.02	0.45±0.03**	0.38±0.023	0.44±0.01**	0.375±0.03	0.42±0.029*
AngII(pg/ml)	42.1±2.4	49.9±8.5*	43.2±4.8	49.4±5.9	40.6±4.2	51.8±6.7**
ΔPV(%)	/	−5.03±3.2	/	−5.84±4.8	/	−7.57±6.3

Data are presented as mean±SD. HR indicates heart rate; SBP, systolic blood pressure; DBP, diastolic blood pressure; NE, norepinephrine; E, epinephrine; ANP, Atrial Natriuretic Peptide; AngII, angiotensinII; PV, plasma volume.

* P<0.05,

** P<0.01,

*** P<0.001 significant difference within group with respect to Before value.

**Figure 1:**
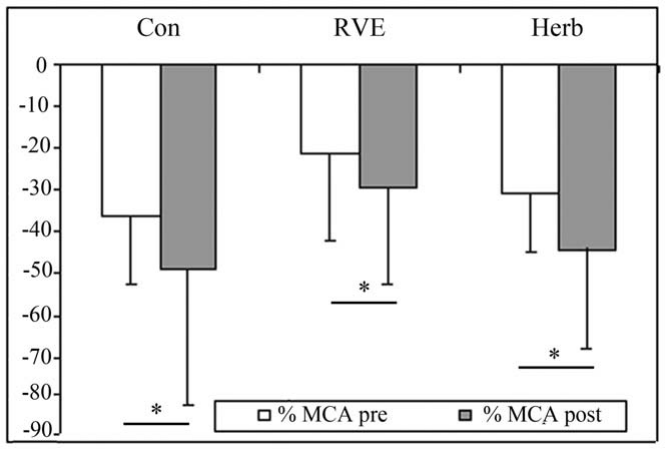
Percentage change in flow velocity in the middle cerebral artery (MCA) from the supine to tilt positions. The MCA was measured by Doppler ultrasonography as detailed in the Materials and Methods section. Percentage change in flow velocity in the MCA from the supine to tilt positions was significantly increased after 60-day HDBR in all groups (P<0.05).

**Figure 2:**
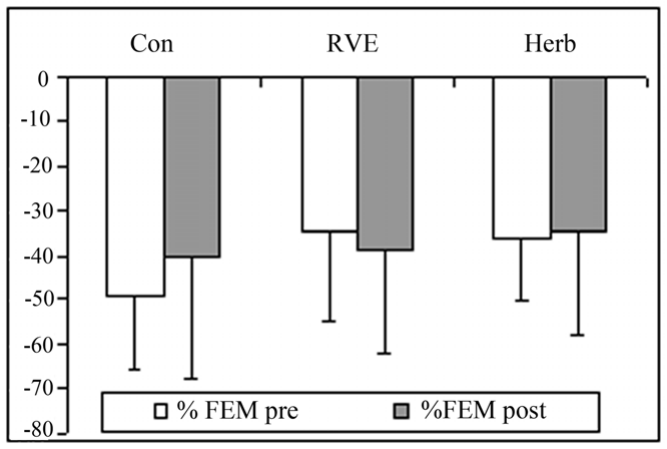
Percentage change in the flow velocity of the femoral artery (FEM) from supine to tilt positions. The FEM was measured by Doppler ultrasonography as detailed in the Materials and Methods section. The percent change in FEM from supine to tilt positions post-HDBR was similar to pre-HDBR in Con, RVE and Herb groups.

**Figure 3:**
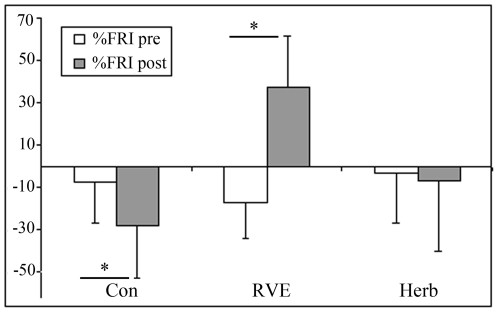
Percentage change in resistance in the femoral artery (FRI) from supine to tilt positions. FRI response decreased in the Con gr (p<0.05), increased in the RVE gr (p<0.05) and was maintained in the Herb gr. RVE and Herb contributed to maintain vasoconstrictive responses at the leg level but the effect of RVE was greater.

**Figure 4:**
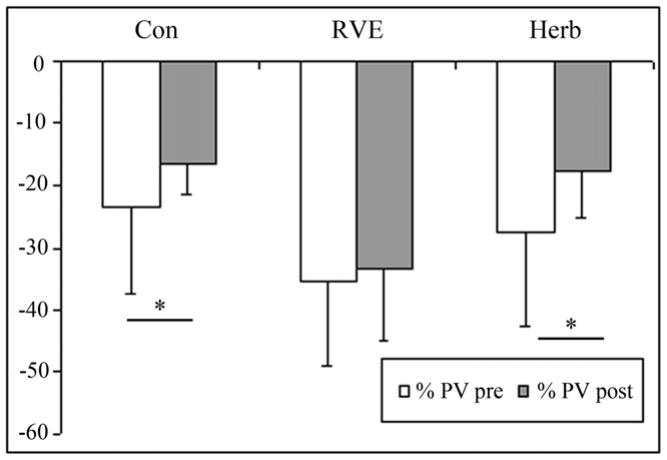
Percentage change in flow in the portal vein flow (PV) from supine to tilt positions. The PV was measured by echography as detailed in the Materials and Methods section. The percent change in the PV from supine to tilt positions decreased less post-HDBR in Con and Herb groups (p<0.05), but there was no change in the RVE gr. RVE contributed to maintain splanchnic vasoconstriction in response to tilt-induced fluid shift.

**Figure 5:**
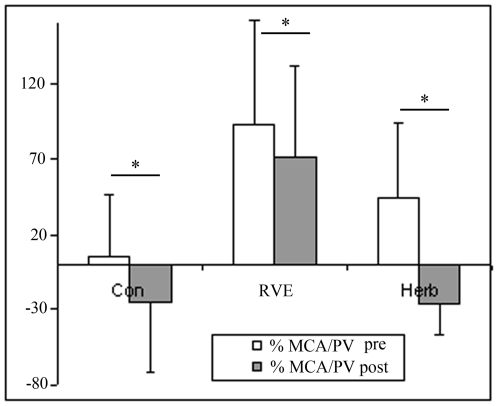
Ratio of flow of the cerebral vein to portal vein (MCA/PV). The cerebral-to-splanchnic flow ratio confirmed that only RVE countermeasures contributed to redistribute the cardiac output in favor of the brain area.

At post-HDBR tilt (Figure 1), MCA decreased more compared with pre-HDBR in the Con and CM gr (Con pre, −36±21% *vs* post, −49±37%; RVE pre, −21±14% *vs* post, −30±6%; Herb pre, −31±20% *vs* post, −44±19%, p<0.05). FEM was reduced to a similar extent in pre-HDBR in the Con gr (pre, −49±16% *vs* post, −41±27%), in RVE gr (pre, −35±21% *vs* post, −39±23%) and in the Herb gr (pre, −36±14% *vs* post, −35±25%, Figure 2). The MCA/FEM tended to decrease in the Con gr (pre, 24.6±49% *vs* post, −21.9±71.8%) and Herb (pre, 11±36% *vs* post, −6.85±23.8%) but remained stable in the RVE gr (pre, 16.67±13% *vs* post: 10.62±27%. FRI dropped in the Con gr (pre, −7.62±19.46% *vs* post, −28.15±26.1%, p<0.05), remained stable in the Herb gr (pre, −3.46±23.35% *vs* post, −6.85±33.39%) and increased in the RVE gr (pre, −17.34±16.8% *vs* post, 37.33±24.39%, p<0.05, Figure 3). PV decreased less in the Con gr (pre, −23±13% *vs* post, −17±4.84%, p<0.05) and Herb gr (pre, −28±15% *vs* post, −18±7%, p<0.05) but remained unchanged in the RVE gr (pre, −36±14% *vs* post, −33±12%, Figure 4). MCA/PV decreased in the Con gr (pre, 5±41% *vs* post, −25±47%, p<0.05) and Herb gr (pre, 44±50% *vs* post, −26±21%, p<0.05), but increased in the RVE gr (pre, 93±69% *vs* post, 71±61%, p<0.05, Figure 5).

**Figure 6:**
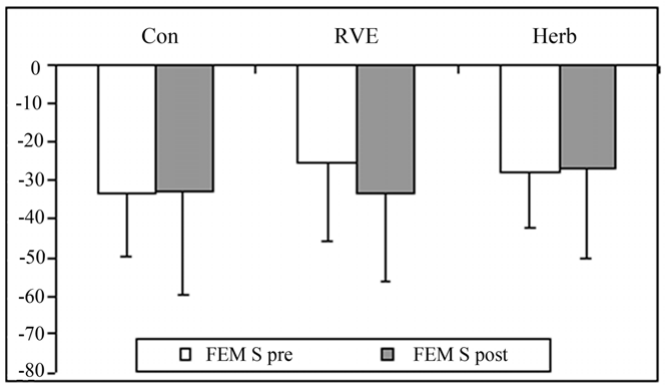
“FEM S” femoral systolic velocity changes were in the same proportion as stroke volume. There was no significant difference in the drop in FEM S (i.e., stroke volume) pre- and post-HDT tilt. Differences in vasomotor responses between the groups were not related to changes in cardiac output. This confirmed that each area provides a specific response that is due to local morphological or functional disturbances.

FEM systolic velocity dropped similarly in all 3 groups during the tilt test (Figure 6), which meant that stroke volume also dropped in all groups [22].

**Figure 7:**
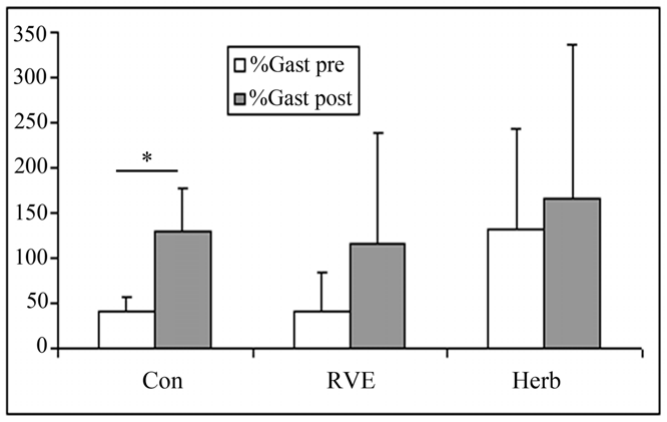
Percentage changes in gastrocnemius (GAST) section from supine to tilt positions. Gast section was measured by echography as detailed in the Materials and Methods section. The percent change in Gast section from supine to tilt positions was increased more post-HDBR in the Con gr. RVE and Herb contributed to limit Gast distension, with the effect of Herb being greater.

**Figure 8:**
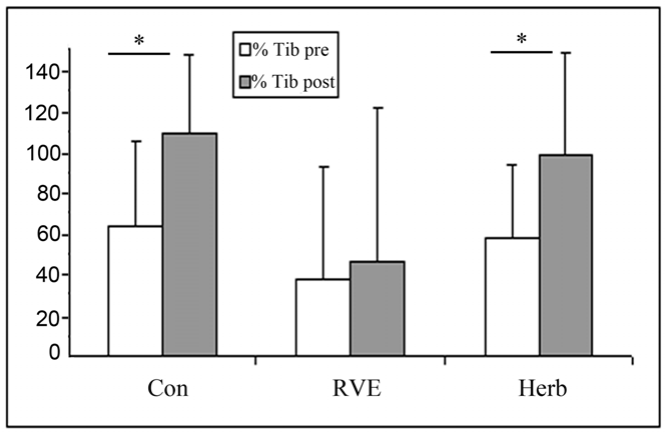
Percentage change in the tibial vein (Tib) section from supine to tilt positions. The Tib section was measured by echography as detailed in the Materials and Methods section. The percent change in Tib section from supine to tilt positions was significantly increased (P<0.05) post-HDBR in Con and Herb groups, but not in the RVE gr. Only RVE CM contributed to maintain vein distension at the pre-HDBR level.

The CSA of Gast increased significantly more in the Con gr only (pre, 40±40% *vs* post, 74±48%, p<0.05, Figure 7) whereas the CSA of the Tib increased more in the Con gr (pre, 63±42% *vs* post, 109±38%, p<0.05) and Herb gr (pre, 58±36% *vs* post, 98±50%, p<0.05) but not in the RVE gr (pre, 37±55% *vs* post, 47±75%, p>0.05, Figure 8). The percentage change in Saph was similar pre- and post-HDBR tilt in the Con, RVE and Herb groups.

The limited proportion of NF subjects (23% of 21 subjects) and the small number of NF in each group (2 Con, 1 RVE, 1 Herb) did not allow study of the impact of the 2 CMs on orthostatic tolerance.

## Discussion

In the Con gr, vasoconstriction was reduced after HDBR in the leg (FRI increased less) and splanchnic areas (PV reduced less; MCA/PV increased less) and consequently the flow redistribution towards the brain became less efficient post-HDBR tilt than at pre-HDBR tilt. These findings are in agreement with those reported by other authors looking at short- and long-term bedrest [3]. Moreover, after 60-day HDBR, RVE contributed to maintain vasoconstriction in splanchnic and lower limb areas, while Herb contributed to maintain vasoconstriction in the lower limb only. Thus, one may consider that these 2 CMs contributed to reduce orthostatic intolerance.

Nevertheless, the daily squat-up period (even of short duration) may also have contributed to partially prevent deterioration of the vasomotor response in the leg and splanchnic areas in all groups, which also helped to maintain orthostatic tolerance [23]. Only 23% of the subjects were NF at post-HDBR tilt, whereas in most of the studies in which subjects remained strictly in a bedrest anti-orthostatic position the proportion of NF was ≈60%. Other studies using a short-radius centrifuge or a short period standing during bedrest reported significant reduction in NF, as in the present study [5], [6].

Even though the daily squat-up period certainly partially reduced the effect of HDBR on the cardiovascular system, the RVE and Herb CMs were found to significantly reduce the deleterious effect of HDBR on the response to the tilt-induced fluid shift. At the lower-limb level, RVE and Herb CMs contributed to keep the vasoconstrictive response against the fluid shift (increased vascular resistance, high drop in femoral flow) whereas the effect of RVE was higher than those of Herb CM.

We previously reported that the lack of vasoconstrictive response induced by HDBR was related to the local abnormal responses of the vasoconstrictive targets [3]. This finding supports the notion that CMs may have a significant impact on distal cardiovascular targets. Plantar vibration induces significant sympathetic activity in lower-limb nerves [24]. Vibration of ≈60 Hz applied to the hand generates a significant increase in local sympathetic nerves and in blood flow to the skin as measured by laser Doppler ultrasonography [9]. Moreover other HDBR studies demonstrated that RVE compensates for the decrease in diameter of leg arteries, the increase in thickness of the walls of the carotid and femoral arteries, and the decrease in vasodilator capacity induced by HDBR [25], [26]. We suggest that, in the present study, daily plantar vibration (part of RVE) triggers a sympathetic response close to the one triggered by orthostatic stress which prevents degradation of the vasomotor process. This notion is supported by the higher increase in FRI in the RVE gr.

RVE also prevented changes in splanchnic vasoconstriction because the reduction of portal flow to tilt after HDBR was not different from pre-HDBR in this group whereas it was less reduced in the Con gr and Herb gr during the post-HDBR tilt test. On the basis of a study which reported that hand–arm vibration can increase sympathetic activity and induce repeated vasoconstriction at the leg level, we suggest that feet vibration may induce vasoconstriction locally as well as in other territories, such as the splanchnic territory [9].

The increase in the CSA of Gast from the supine to tilt position was higher post-HDBR in the Con gr whereas there was no significant difference in the RVE gr and Herb gr between pre-and post-HDBR. RVE and Herb contributed to reduce distension in the Gast during tilt after HDBR, but the effect of Herb was greater. Cross-sectional distension in the Tib from the supine to tilt position was also greater post-HDBR except in the RVE gr. Thus, only the RVE CM contributed to maintain the distension in the Tib at the pre-HDBR level. A low-level plantar vibration (30–60 Hz) was found to significantly prevent the drop in BP induced by the orthostatic test [10]. The author suggested that the plantar vibration may stimulate type-IIA muscle fiber activity in the leg, which is critical for effective skeletal muscle pumping in the absence of locomotion. The muscle component may help to reduce vein distension in the standing position. Another long-term (52 day) HDBR reported that RVE had no effect on the diameter, capacitance or compliance of veins [27]. That finding did not contradict our results because the measurements were done at rest and not during the tilt test.

One may suggest that the Gast inserted into the calf muscle is more protected than the Tib, which is free of muscle constraint and does not face the same effects during HDBR. Conversely, we should be more concerned by muscle atrophy, which occurs during bedrest. However, a recent bedrest study demonstrated that the Gast was not particularly sensitive to muscle atrophy [4]. Thus, the RVE CM contributed to limit the maximal distension to Tib and Gast by stimulating venous return thanks to muscle contraction, which may keep the vein response to fluid shift from the plantar area operational.

TKYXCHP significantly attenuates the decrease in left ventricular diastolic volume (LVDV) and stroke volume (SV) induced by 28-day hindlimb unloading in rats [28]. Moreover, during this 60-day HDBR, measurement of thoracic impedance showed that the 60-day HDBR induced reduction of the cardiac pump and systolic function, which were significantly improved by TKYXCHP [29]. These results are in accordance with our findings by echography: after 58-day HDBR, LVDV decreased 9.75% in the Con gr, 5.72% in the RVE gr and 5.14% in the Herb gr; SV decreased 16.34% in the Con gr, 11.8% in the RVE gr, and 8.15% in the Herb gr [23]. As the plasma volume(estimated from the left ventricle volume) was reduced in all gr but not as much as usually found in bedrest studies, we suggest that the 10 min standing prevented partially the hypovolemia usually observed in strict bedrest in all 3 gr. Magrini et al [30] evaluated the cardiovascular effects of gravity in healthy males before(6 months) and after(18 months)acquiring the ability to stand, finding that the erect posture represents a phase when, for the first time in the natural history of the cardiovascular system, translocation of intravascular volume from the cardiopulmonary area to the periphery stimulates nervous and humoral responses to control the dynamics of body fluids and arterial blood pressure in gravitational environment. In this study, 10 min standing exercised the control ability of the dynamics of body fluids, which maybe readapt to the long-term HDBR. Moreover, the feet shift of intravascular volume during standing may reduce the release of ANP and alleviate the decrease of plasma volume induced by HDBR. Besides the protection of cardiac function in HDBR, Herb was expected to have a potential role in maintaining vascular functions. Among ≈500 Chinese herbs currently used, several herbs have primary cardiovascular indications [31]. Most of these herbs are used in China based on their pharmacological mechanisms of dilating cardiocerebral vessels, suppressing platelet aggregation, improving the circulation, removing blood stasis, protecting against ischemic–reperfusion injury, and enhancing the tolerance of ischemic tissue to hypoxia [13]. The NO–NOS pathway is the main target of these TCMs [31]. Some active ingredients have been found in Chinese herbs. For example, tetramethylpyrazine (TMP) from *Ligusticum wallichii* (one of the main ingredients of TKYXCHP) is known for its cardiovascular protective effects [32]. However, the present study showed that Herb CM partially protected the peripheral artery and venous responses to the tilt test after 60-d HDBR (maintenance of FRI and limitation of increase in Gast CSA during the tilt test compared with control). For the first time, the Herb CM was found to have beneficial effects on lower-limb vasoconstrictive responses to orthostatic stress. Most studies on the effects of Chinese herbs on the vascular system focus mainly on the endothelium and vasorelaxation at rest: there are few reports about their effects on vasoconstriction during dynamic tests. Of the main ingredients of TKYXCHP, only Panax ginseng is thought to have a potential role of elevating blood pressure [33], which may have direct effects on vascular smooth muscle. Herb CM had obvious effects only on lower-limb areas, not in splanchnic areas. Hence, we can suppose that Herb CM may protect the mechanical properties and functionality of the distal arterial and venous network. Another 60-day bedrest test reported that, after 55-day bedrest, the vasoconstrictive response to LBNP was reduced in the control but not in the exercise group, whereas in both cases the sympathetic response (as measured by peroneal nerve micro-neurography) was not affected [3]. It was concluded that the exercise CM prevented degradation of neurotransmission at the level of the distal vessels. Moreover, lower-limb vascular resistance (FRI) at rest was not altered by bedrest in the control or exercise groups. This finding confirmed that at-rest status does not necessarily interfere with the dynamic vasoconstrictive response to the tilt test. Such observations support the notion that Chinese herbs should protect not only vessel status at rest (NO/NOS pathway) but also the distal neurotransmission process. The vascular changes caused by TKYXCHP could help us to understand how herbs protect against adverse cardiovascular effects of simulated microgravity or spaceflight.

Changes in femoral systolic peak velocity (FEM S) were similar in the three groups, so the changes in SV were also similar [22]. Thus, neither of the 2 CMs had a significant impact on the change in FEM S (i.e., stroke volume) during the tilt test even though RVE and Herb contributed to reduce the loss of left ventricular volume. Therefore, the difference in vasomotor responses in the RVE gr and Herb gr compared with the Con gr could not be related to a change in cardiac response nor to hypovolemia but instead to changes in the vasoconstrictive response in the different distal areas in relation to local morphological or functional changes, as suggested above.

### Conclusion

(a) RVE and Herb contributed to protect the reactivity of the vascular system because the vascular response to the tilt test in these groups was better maintained than in the Con gr; (b) RVE had a stronger impact than Herb on the cardiovascular response to tilt after HDBR.
